# Fabrication of Fe-Al nanoparticles by selective oxidation of Fe-Al thin films

**DOI:** 10.1186/1556-276X-8-152

**Published:** 2013-04-02

**Authors:** Pyungwoo Jang, Seungchan Shin, Chip-Sup Jung, Kwang-Ho Kim, Kyu Seomoon

**Affiliations:** 1 Department of Optical Engineering, Cheongju University, Cheongju, 360-764, South Korea; 2 Department of Semiconductor Engineering, Cheongju University, Cheongju, 360-764, South Korea; 3 Department of Applied Chemistry, Cheongju University, Cheongju, 360-764, South Korea

**Keywords:** Selective oxidation of aluminum, Particulate Fe-Al films, Fe-Al nanoparticles, Water vapor, Hydrogen, 81.07.Bc, 75.70-I, 75.75.Fk

## Abstract

The possibility of a new technique for fabricating nanoparticles from thin films using selective oxidation in an atmosphere mixture of water vapor and hydrogen was investigated. Fe-5wt.%Al films were RF-sputtered and annealed in the atmosphere mixture at 900°C for up to 200 min, in order to oxidize aluminum selectively. Thermodynamics simulation showed that temperatures exceeding 800°C are necessary to prevent iron from being oxidized, as confirmed by the depth profile of XPS. As the annealing time increased, the morphology of the 200-nm Fe-Al films changed from the continuous to the discontinuous type; thus, particulate Fe-Al films formed after 100 min. The particulate 10- to 100-nm Fe-Al films showed super-paramagnetic behavior after the oxidation. Thus, a new technique for fabricating nanoparticles was successfully introduced using selective oxidation.

## Background

Aluminum oxide, Al_2_O_3_, formed on the surface can be used as a mechanically protective, oxidation-resistive, electricity-insulating film. For example, it was reported that in Fe-Al-X bulk alloys, the aluminum elements out-diffused along the *α*-Al_2_O_3_ grain boundary formed in an alumina network on the boundary by the selective oxidation of aluminum when the alloys were annealed in the atmosphere [1]. The Al_2_O_3_ layer could also be formed exclusively by selective oxidation when Fe-Al alloys were annealed in a mixed atmosphere of water vapor and hydrogen at elevated temperatures [2,3]. It was also reported that the Fe depletion zone appeared in the annealed Fe films on an Al_2_O_3_ substrate [4]. This indicates that continuous Fe films changed to discontinuous films, i.e., particulate films. However, they did not focus on the morphological change of the Fe/Al_2_O_3_ films, nor the reasons for it. It is interesting to investigate the morphological changes and related properties of Al_2_O_3_/Fe-Al films, in which oxide is formed on the surface of Fe-Al films by the selective oxidation of aluminum in Fe-Al films in a mixed atmosphere.

In this study, morphological change, as well as analyses of the chemical, structural, and magnetic properties of selectively oxidized Fe-Al films formed on SiO_2_ substrates are investigated by X-ray photoelectron spectroscopy (XPS), scanning electron microscope (SEM), transmission electron microscope (TEM), and vibrating sample magnetometer (VSM), with a special emphasis on the possibility that nanoparticles in the shape of a sphere can be formed by the selective oxidation of aluminum in Fe-Al films.

## Methods

**Figure 1:**
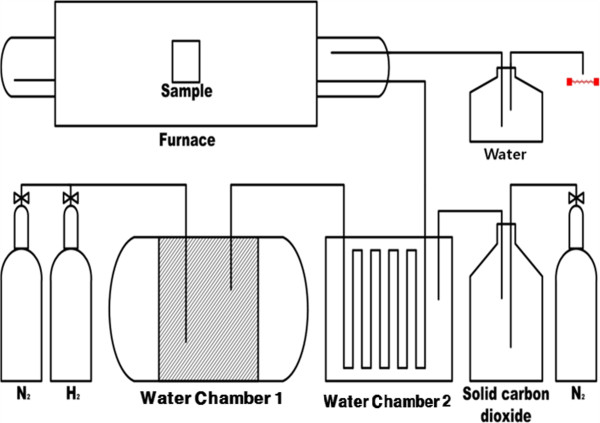
**Schematic diagram of the experimental setup for selective oxidation of Fe**-**Al films.**

The STANJAN program was used to determine the optimum annealing conditions for the selective oxidation of aluminum in Fe-Al films [5]. For this, 10- to 200-nm-thick Fe-5wt.%Al films were radio frequency (RF) sputtered from a 4-inch Fe-5wt.%Al alloy target at room temperature on 100-nm-thick SiO_2_ substrates, for which the Si wafers had been oxidized at 1,000°C for 110 min. The sputtering pressure, input power, and Ar flow rate were 5 mTorr, 100 W, and 10 sccm, respectively. As-sputtered films were annealed at 900°C for up to 200 min in an atmosphere mixture of water vapor and hydrogen, for which hydrogen passed through a copper pipe whose temperature was kept at -17°C at flow rates of 500 and 1,800 sccm (Figure 1). The concentration of the water vapor in the atmosphere mixture was controlled by adjusting the temperature of water chamber 2. At -17°C, the vapor pressure of water is about 1 Torr [6]. The magnetic properties of the films were measured using a VSM. The surface morphology and composition were analyzed using SEM (VEGA/SBH, TESCAN, Brno-Kohoutovice, Czech Republic) with an energy dispersive X-ray spectrometer (EDS) attachment. Cross-sectional images of the particulate films were observed using TEM (JEM-2100F, JEOL Ltd., Akishima-shi, Japan). Variations of chemical state and the composition with film depth were analyzed using XPS with Mg_*Kα*_ radiation.

## Results and discussion

**Figure 2:**
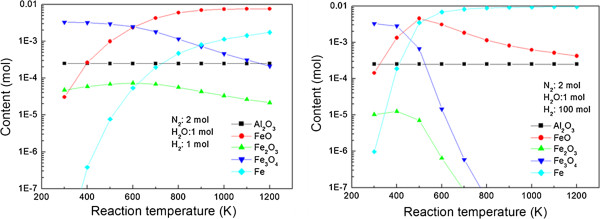
Dependence of product content on reaction temperature simulated by the STANJAN program.

Because the standard molar enthalpies of the formation of Al_2_O_3_, FeO, Fe_2_O_3_, and Fe_3_O_4_ at 298.15 K are -1675.7, -272, -824, and -1118.4 kJ/mol, respectively, it can be inferred that Al_2_O_3_ is preferentially formed under oxidation conditions. To confirm this, as well as in order to simulate the optimum conditions for the selective oxidation of aluminum, the STANJAN program was used. Figure 2 shows the simulation results of the reaction temperature versus the product content, with the input amounts of Fe, Al, H_2_O, and H_2_ given as 0.01, 5×10^-4^, 1, and 1 mol (left figure), and 100 mol (right), respectively. Al_2_O_3_ is formed exclusively at all temperatures. In addition, Fe_3_O_4_ is dominant at lower temperatures, while the formation of iron oxides is hampered with increasing temperatures; therefore, temperatures exceeding 800°C were considered ideal for the selective oxidation of aluminum. However, if the hydrogen content is not enough, formation of FeO is expedited even at a high temperature. When the ratio of hydrogen and water vapor content is 1:1, FeO is dominant at a high temperature, as shown in the left-hand figure of Figure 2.

**Figure 3:**
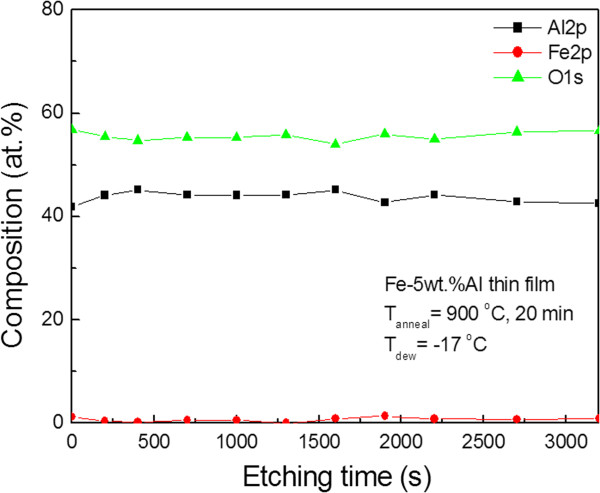
**XPS depth profile of Fe-Al film oxidized for 20 min at 900°C.** (*T*_Anneal_ = 900°C, *T*_Dew_ = -17°C, and *t* = 20 min).

**Figure 4:**
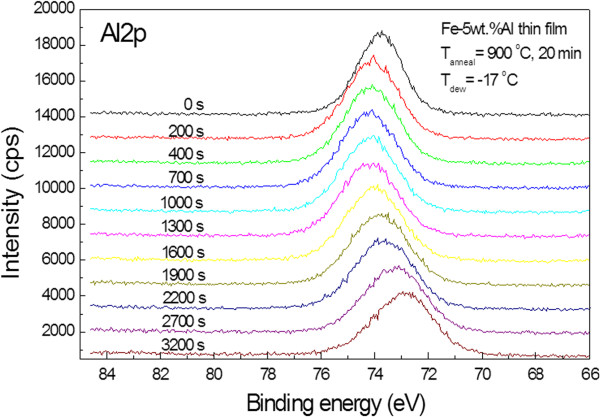
Variation of binding energy of aluminum Al2p state with depth of the Fe-Al film oxidized selectively.

Selective oxidation of aluminum was also confirmed by the XPS depth results of post-oxidized Fe-Al films. Figures 3 and 4 show the XPS compositional depth profile and the variation in aluminum Al2p binding energies with depth, respectively, when the Fe-Al film was annealed for 20 min at 900°C. Iron is not detected until 3,200 s, while the content ratio of aluminum to oxygen is approximately 2:3, which means that Al_2_O_3_ is formed on the surface of the film. The Al_2_O_3_ layer was assumed to be thicker than 50 nm because the etching rate during XPS depth profiling was approximately 1 nm/min. From the fact that the binding energies of aluminum in metallic aluminum and in aluminum oxide (Al_2_O_3_) are 73 and 74.3 eV, respectively, Al_2_O_3_ is formed on the top surface of the film. Also, it can be inferred that the oxide thickness is about 53 nm because metallic aluminum is not detected until 3,200 s after etching. It was reported that *γ*-Al_2_O_3_ is formed when Fe-5wt.%Al bulk alloy is annealed in the atmosphere mixture at a temperature higher than 920°C [3]. However, peaks diffracted from the (110), (200), and (211) plane of *α*-Fe were found in the XRD experiment. No peak from aluminum oxide was found.

**Figure 5:**
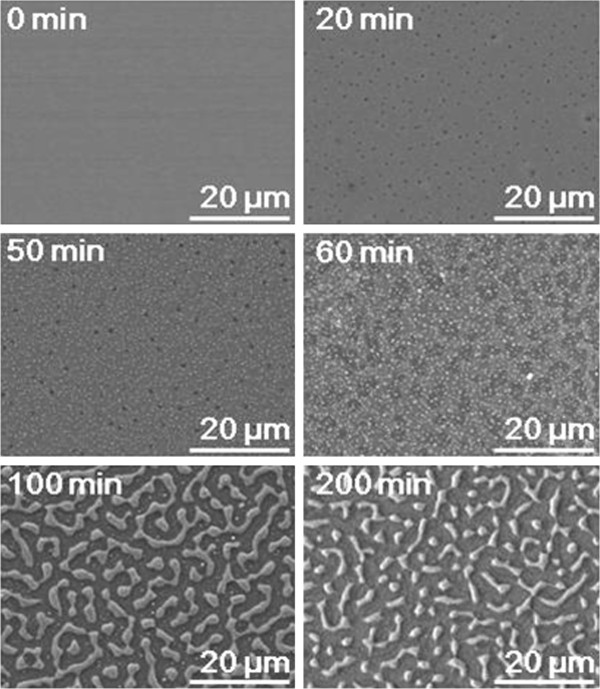
SEM surface morphology of 200 nm Fe-Al films oxidized selectively.

SEM analysis was conducted (Figure 5) with films that were oxidized for up to 200 min at 900°C, with a hydrogen flow rate of 500 sccm and a dew point of -17°C. Very small, white and black dots were observed after 20 min of oxidation. After 50 min, the dots became larger, and after 60 min, the black dots became substantially larger, as well as irregular. The gray particles corresponding to oxidation for 20, 50, and 60 min indicate a continuous Fe-Al film. After 100 min, the Fe-Al film became discontinuous and particulate. After 200 min, the particles of the Fe-Al film became completely disconnected. The compositions of the three particle types, that is, large particles, small particles, and black particles of films oxidized for 50 min, were analyzed using EDS. The Al compositions of the white, gray, and black particles were 5.6, 8.8, and 33.5 wt.%, respectively. The Fe, Al, and O compositions of the large and white particles of the 200-min-annealed film were 90.8, 4.5, and 4.8 wt.%, respectively, while those of the black region were 19, 33.6, and 47.4 wt.%, respectively. Therefore, it was inferred that the large and small white particles are Fe-Al alloy grains covered by Al_2_O_3_. However, the mechanism by which these different Fe-Al particles were formed differed. The small particles were formed in an early stage of oxidation and then grew through Ostwald ripening. In contrast, the large particles were formed by the growth of the black dots, which were holes. If holes are formed and grown in the films, the films will contract and become discontinuous. The contraction or shrinkage of the film and the growth of the holes reduce the interfacial energy. However, it seems that the Fe-Al films become particulate at a faster rate only when the films are annealed in the mixed atmosphere. If the films are annealed at 900°C for 200 min in an atmosphere with a very low dew point of -196°C (liquid nitrogen's temperature), the films do not become particulate. No equilibrium vapor pressure at -196°C has been reported yet. However, the equilibrium vapor pressure at -196°C can be inferred to be extremely low, from the fact that the equilibrium vapor pressure at -80°C is reported to be 0.055 Pa (4.12×10^-4^ Torr) [6].

**Figure 6:**
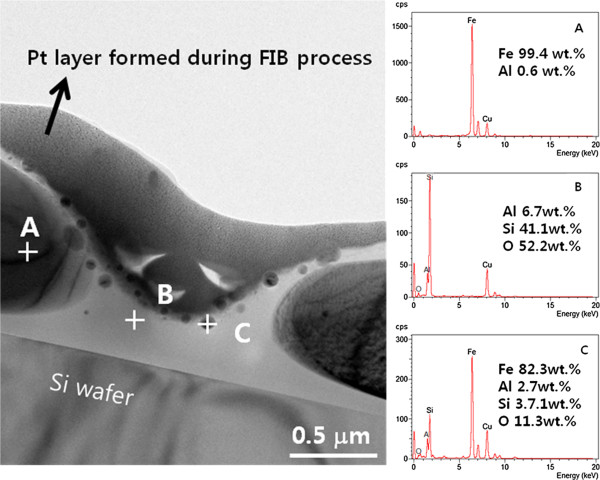
Cross-sectional TEM image and EDS results of Fe-Al film oxidized selectively.

TEM cross-sectional analysis was also done, as shown in Figure 6. The film was oxidized for 200 min at 900°C, with a hydrogen flow rate of 500 sccm and a dew point of -17°C. The large black particles (A), white region (B), and small black particles (C) in Figure 6 correspond to the large white particles, black region, and small white particles, respectively, in the SEM image of the 200-min-annealed Fe-Al film shown in Figure 5. Contrary to the EDS analysis of SEM, in which the depth of the affected zone stimulated by incident electrons is several micrometers, the affected zone in the EDS analysis of TEM is very thin. The large particles (A) are nearly pure iron, while the oxide layer (B) contains lots of silicon and small particles. The small black particles (C) also contain several weight percentages of silicon. Silicon is detected because of the large difference in the standard enthalpy of formation between SiO_2_ and Al_2_O_3_, as shown below.

$$\begin{array}{ll} 2\;\mathrm{Al}+\frac{3}{2}O_{2}\to {\mathrm{Al}}_{2}\;O_{3} & \Delta H_{o}=-1,675.7\;\mathrm{kJ}/\mathrm{mol}\\ \mathrm{Si}+O_{2}\to {\mathrm{SiO}}_{2} & \Delta H_{o}=-877\;\mathrm{kJ}/\mathrm{mol}\\ 3\;{\mathrm{SiO}}_{2}+4\mathrm{Al}\to 3\;\mathrm{Si}+2{\mathrm{Al}}_{2}O_{3} & \Delta H_{o}=-720.4\;\mathrm{kJ} \end{array}$$

Therefore, silicon dioxide in contact with the Fe-Al film is reduced to silicon while the metallic aluminum in the Fe-Al films is oxidized into Al_2_O_3_. The silicon created diffuses out while the Al_2_O_3_ diffuses into SiO_2_ and forms a mullite (3Al_2_O_3_·2SiO_2_). Thus, the SiO_2_ layer transforms into a mixture of mullite and SiO_2_. The out-diffused silicon can be dissolved into small Fe-Al particles, which are formed in an early stage of oxidation. The reason for non-detection of Si in large particles is not clear yet.

**Figure 7:**
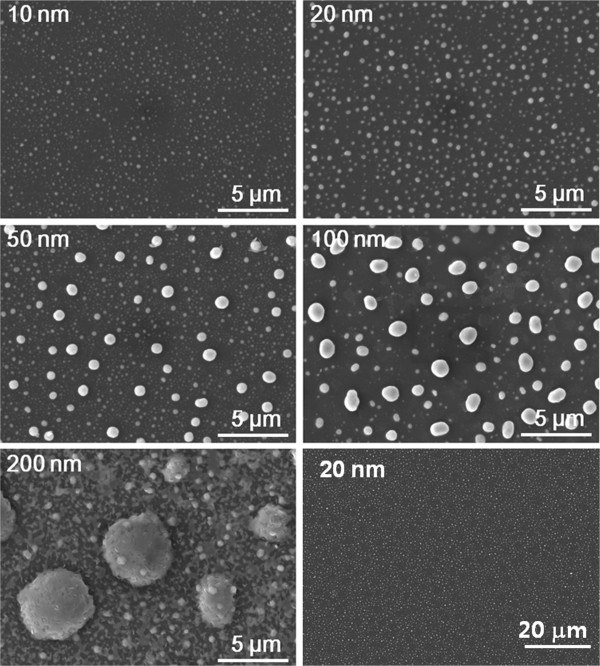
SEM images of 10 to 200 nm Fe-Al films selectively oxidized at 900°C for 200 min.

The particles shown in Figure 5 are too large to exhibit the properties of nanoparticles. The 10 to 100 nm Fe-Al films were RF-sputtered and then annealed for 200 min at 900°C, with a hydrogen flow rate of 500 sccm and a dew point of 0°C. As shown in Figure 7, the films also become particulate after oxidation. The thinner the films become, the smaller the particles become. In addition, particle sizes were not uniformed, and their shape is rather spherical. Moreover, black holes found in the films oxidized for 20 to 60 min can be seen in Figure 5: they are clearly observable at lower magnification (right lower photo). In the black region, very small particles are found. It seems that the white particles are Fe-Al particles, which are very similar to the small particles formed in the early stage of oxidation shown in Figure 5. From the fact that there are not many small particles near larger particles in the 50-nm-thick film, Ostwald ripening is promoted by the increasing film thickness. In the 200-nm-thick film, the particles have a spherical shape, which is very different from the maze-like shape in the films shown in Figure 5, which were oxidized at an atmosphere with a lower dew point. Maximum particle sizes of the 10-nm- and 20-nm-thick films are about 0.3 and 0.47 μm, respectively. The minimum particle size in the 20-nm-thick films is smaller than one-tenth of the maximum size.

**Figure 8:**
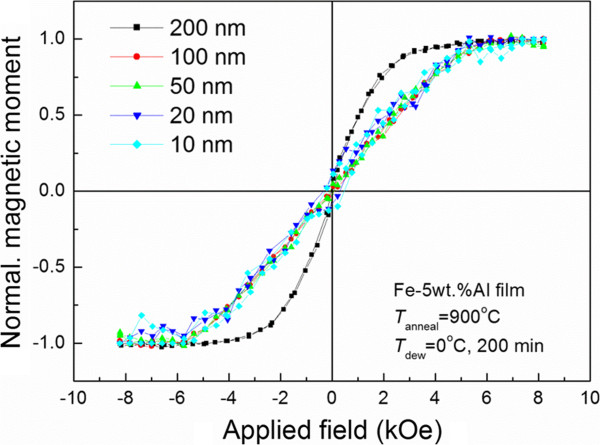
Normalized VSM loops of 10 to 200 nm Fe-Al films selectively oxidized at 900°C for 200 min.

When the Fe-Al films were selectively oxidized, the slope of the hysteresis loops at the origin decreased, due to the demagnetization field, as the oxidation time increased. Figure 8 shows normalized VSM loops of the Fe-Al films of Figure 7 measured at room temperature. The slope of the magnetization curve of the as-sputtered Fe-Al film was very high near the origin. Further, it decreased gradually as oxidation time increased. The 200-nm-thick film shows hysteresis, while the other films do not show hysteresis. Moreover, the normalized loops of the 10- to 100-nm-thick films have nearly same slope and shape, which means that these particles are superparamagnetic at room temperature. Because magnetocrystalline easy axis and the magnetocrystalline anisotropy energy of iron are <100> and *K*_1_ = 4.8×10^4^ J/m^3^, respectively, superparamagnetic behavior appears, even though the maximum particle size is about 1 μm, which is very much larger than materials with uniaxial crystalline anisotropy.

## Conclusions

The 10- to 200-nm-thick RF-sputtered Fe-Al films were oxidized in the atmosphere mixture at 900°C for up to 200 min. Small particles formed in the early stage of oxidation while large particles formed by contraction of the film or the growth of black holes. Formation of Al_2_O_3_ on the surface of the film was confirmed by both the depth profile and chemical shift of the Al2p state upon XPS analysis. The 10- to 100-nm-thick films after oxidation showed superparamagnetic behavior that was due to Fe-Al nanoparticles. Thus, a new technique for fabricating nanoparticles by selective oxidation has been successfully introduced.

## Competing interests

The authors declare that they have no competing interests.

## Authors’ contributions

PWJ is in charge of this project and designed it. SCS carried out most of the experiment including deposition, oxidation, and VSM measurement. CSJ and KHK provided thin film deposition and analysis technique. KS analyzed the XPS results. All authors read and approved the final manuscript.
